# Making the Case for Multi-Axis Assessment of Behavioural Problems

**DOI:** 10.3390/ani10030383

**Published:** 2020-02-27

**Authors:** Jaume Fatjó, Jonathan Bowen

**Affiliations:** 1 Chair Affinity Foundation Animals and Health, Department of Psychiatry and Forensic Medicine, School of Medicine, Autonomous University of Barcelona, 08193 Bellaterra (Barcelona), Spain; 2 Queen Mother Hospital for Small Animals, Royal Veterinary College, Hawkshead Lane, North Mymms, Hertfordshire AL9 7TA, UK; jbowen@rvc.ac.uk

**Keywords:** companion animal, behaviour problem, mental health

## Abstract

**Simple Summary:**

Companion animals can experience behavioural and mental health problems that are similar to those we see in people. These problems are influenced by many factors, including an animal’s genetic background, its rearing environment, the environment it lives in, and the social relationships it has with people and other animals. This paper proposes a model for collecting and organising information about all of the factors that contribute to behavioural and mental health problems in companion animals, in order to produce a comprehensive, structured assessment of them.

**Abstract:**

The systematic classification of human mental health disorders and behavioural problems in companion animals face the same challenges. These disorders and problems are complex, multi-factorial, and can interfere with the individual’s ability to function within society, a social or family environment. Classification systems are reductive, they discard a lot of critical information, and can be overly focused on the presenting problem, inflexible and obstructive to new research. As a result, human psychiatry is moving away from classification systems and toward a clinical and research model based on dimensional characteristics that encompass the full range from normal to abnormal, and include multiple sources of influence from genetic, to environmental and psychosocial. In this paper, we set out a multi-axis model for the collection and organisation of information about companion animal behaviour problem cases that avoids some of the limitations of classification systems, is aligned with the current research approach in human psychiatry, and assists the clinician in making a complete and thorough assessment of a case.

## 1. Introduction

The systematic description and classification of behaviour problems is an unresolved issue in companion animal behavioural medicine [1]. Any classification system faces three fundamental challenges:

- Behaviour is a complex construct that is resistant to classification using discrete labels.
- There is an ongoing debate about whether behaviour problems should be considered normal adaptive responses or dysfunctional conditions.
- There are discrepancies between authors on the nature, associated risk factors, and clinical presentation of many behaviour problems.

Classification is a process by which complexity is reduced and diagnosis is organised into a series of discrete categories. This solution is appropriate for those behavioural problems for which a clear causal neurophysiological or neuropathological process has been identified. Canine cognitive dysfunction is a good example, although it should be remembered that it is currently a diagnosis of exclusion that, similar to human Alzheimer’s disease, can only be fully confirmed post-mortem.

Most authors in the field of behavioural medicine use some system of classification of behavioural problems, based on commonly occurring constellations of signs that form syndromes. Diagnostic categories usually combine information about (1) the underlying motivation or affective state associated with the problem and (2) triggering stimuli and contextual cues, (3) aetiological factors (where such information is available). Examples include separation anxiety, territorial aggression, defensive aggression, and noise phobia. However, there is little agreement on the classification system; for example, in the area of canine aggression, the number of diagnostic categories suggested by leading authors in current textbooks and reviews varies between 9 and 15, with some categories being absent from some systems and disagreement on the nature of those categories that are more common [2].

The lack of agreement on classification reflects the heterogeneity of presenting signs and contributory factors. Individuals with the same syndromic diagnosis can present in different ways and have very different balances of temperamental, environmental, experiential, and other factors. Neither the aetiology of these syndromes, nor their biology, are properly understood. For example, there is still a fundamental debate about the underlying motivation for family-directed aggression in dogs [3]. There is also a concern that when a diagnostic category is applied, we may lose sight of the uniqueness of that individual [4].

A standardised way to approach behaviour problems would provide many benefits, including better communication between practitioners and a more structured way to conduct research and teaching [1]. However, it should be flexible enough to take into account the natural variability of behaviour, and to include all the factors that interplay in its expression and to embrace the theoretical differences found in the literature [1,2]. It should also take into account the various contributory factors to the problem, and the reasons for its current presentation in the clinic, from the animal’s health to its relationship with the family.

We propose that, rather than focus on classification, an alternative solution is a multi-axis system that captures the full range of information about a case and does not depend on rigid classification. The term “axis” has been chosen to present the different parts of this behavioural assessment model because it is a term that is already established in the human psychiatric field, and it cannot be confused with the research “domains” of the NIMH’s RDoC programme, which we will discuss later. This system needs to be adaptable to a range of companion animal species, and flexible enough to take account of the varying degree of influence each axis will have on behaviour in different species.

The five axes in the present model are:

1. Behaviour:
  This axis would include all the details of the problem behaviour, which could be used to form a descriptive diagnosis.
2. Traits:
  This axis would include information about behavioural, temperamental, and personality traits (which can be considered to be transdiagnostic characteristics), that have been derived from the behavioural history and from relevant psychometric tools or behaviour tests.
3. Health:
  This axis would include a review of the animal’s past and current health, to evaluate the contribution to the animal’s behaviour.
4. Environment:
  This axis would cover the animal’s environment and its contribution to the animal’s behaviour.
5. Functioning:
  This axis would include information about the animal’s ability to function, in terms of its own welfare, its function as a pet, and its potential impact on society. This includes the bidirectional relationship between the behaviour problem on the quality of life of the pet and the family.

The purpose of this paper is to summarise the 5-axis model, to present some of the supporting evidence for each of the five axes, and to discuss how the model could be applied.

## 2. The 5-Axis Model

### 2.1. Axis 1: Behaviour

The aim of Axis 1 is to provide a detailed description of the animal’s behaviour, both as it relates to the presenting problems and in general. Cases are presented with a problem list that is usually drawn up by the owner. This reflects the behavioural issues that the owner finds alarming or intolerable, but may overlook issues that clinicians might regard as important.

Tools such as the Canine Behavioral Assessment and Research Questionnaire [5] (C- BARQ) and the Feline Behavioral Assessment and Research Questionnaire [6] (Fe-BARQ) can be used to gain an overall picture of a case that is less biased by the owner’s concerns or priorities, and can help to identify problems of significance that are not part of the presenting problems list. Questionnaires of this type can be combined with general questions about the animal’s background and lifestyle to provide an initial summary of the situation.

For each presenting problem, or problem of significance, the following information should be gathered, based on a detailed description of incidents:

- The complete behavioural sequence in a wide range of incidents of the problem behaviour.
- The targets, contexts and triggering stimuli, events and interactions for the behaviour.
- The animal’s emotional state, before, during and after incidents.
- The animal’s level of awareness (which relates to its alertness, attention, and visuospatial functioning), during incidents.
- Signaling used by the animal before, during, and after an incident.
- Indications of the motivation or goals of the behaviour.
- The owner’s reaction to the behaviour.
- The evolution of the pattern of behaviour over time.
- Significant events in the animal’s history (developmental environment, relinquishment).
- Information about bite severity (where relevant).

A timeline can be used to record information about the progression of the problem and key events in the animal’s history (such as change of ownership, change of residence, and neutering). This should include information about the dog’s reaction to similar situations to those in which the problem currently occurs (e.g., to identify signs of fear or anxiety without aggression) during each of the animal’s developmental stages before adulthood. For example, how the dog reacted to the presence of an unfamiliar person during the juvenile, adolescent, and adult stages of development.

The assessment of the behavioural axis should enable us to:

- Fully characterise the behaviour and its evolution over time.
- Assess the predictability of the behaviour.
- Make an initial risk assessment.
- Provide information that is required for an assessment of Axis 2 (the evaluation of traits).

For administrative purposes, the pattern of problem behaviour(s) may fit with, and therefore be named as, one or more of the disorder syndromes present in the current literature, but for clinical purposes the emphasis should remain at the descriptive level in order to avoid falsely categorising a condition.

### 2.2. Axis 2: Traits

The objective for the Axis 2 assessment is to identify, and, if possible, quantify any underlying traits which could influence the expression of those axis 1 behavioural responses. A trait can be defined as “a particular characteristic that can produce a particular type of behaviour” (Cambridge English dictionary). This is the broad definition that we have adopted, because it includes low-order (behavioural and temperamental) traits, high-order (personality) traits, coping styles, transdiagnostic traits, and other similar characteristics that have a pervasive, persistent influence on the typical responses of an individual. We have adopted this broad approach because there is currently no consensus on the demarcation between these different types of traits in veterinary species.

Traits reflect relatively stable individual differences in cognitive, emotional, and regulatory aspects of behaviour. They influence behaviour by introducing a bias toward a particular kind of response to a situation. The nature of that bias, its complexity, and its strength will determine the effect that it has. When considering traits, we should consider the ways in which they might contribute to the problem behaviour, as well as the ways in which they might mitigate it.

Traits are also hierarchical in nature [7]. There are low order traits, such as anxiousness, impulsiveness, excitability, sociability, boldness or assertiveness, that are quite narrow in focus and more closely related to neurobiological systems. Some of these low order traits covary in some species, leading to higher order traits such as extraversion or neuroticism. In animals, the distinction between low and high order traits is probably more difficult to make because the difference in complexity between the lowest and highest order traits in, for example, a dog, are much smaller than in a person. This reflects differences in cognitive capability. For some authors temperament is used to describe behavioural consistencies that appear early in life, whereas personality traits only emerge later in life, as temperament interplays with environmental factors and experience [7].

From a clinical perspective, focusing on traits has many potential benefits. Firstly, traits may reflect underlying relatively stable individual differences in biological systems, which could be the target of biological therapies, including drugs, synthetic pheromones, and dietary modification or supplementation. An example of this is the use of fluoxetine to treat problems of aggression in dogs where there is also a lack of impulse control; the individual’s inability to withhold the aggressive response is the primary target of the drug [8]. Secondly, trait assessment could enable us to make predictions about the animal’s likely behaviour in a wide range of situations; for example, when it is faced with a novel or potentially stressful situation.

Within Axis 2, the assessment of traits involves two processes:

- Informal evaluation of traits, versus states, using information from the behavioural history collected in Axis 1.
- Detection and quantification of traits using psychometric or behavioural testing tools.

Stability across time and between different contexts are the key characteristics of a trait that can be identified from the behavioural history obtained in Axis 1, whereas states are transient or situational.

In human psychiatry, traits and states are measured separately using specific psychometric tools.

Another example of a readily identified and important trait that can influence many areas of behaviour is excitability. Excitability is a fundamental characteristic of emotional responses. In both humans and dogs, high levels of arousal seem to impair top-down cognitive inhibition, which in turn has been linked to anxiety and impulse control. For example, in a study of the effects of arousal on inhibitory control, dogs scored worse for inhibitory control failure in a barrier detour task when they were excessively excited [9]. Therefore, excitability could be an underlying factor in a wide range of behaviour problems through its impact on attention, cognition, and emotional intensity. Excitability has also recently been found to be associated with joint hypermobility in dogs, indicating that this trait may be influenced by, or share a common genetic or biological basis with, bodily processes outside of the central nervous system [10].

For some of the traits that are informally identified using information from Axis 1, there may already be questionnaires or behavioural tests that can then be used to quantify those traits, or to confirm their presence. For example, the DIAS impulsivity scale [11] or the ‘excitability’ subscale of the C-BARQ. Although there are fewer available tools for measuring behavioural traits, temperament and personality in veterinary behavioural medicine than human psychiatry, this is an area of active development.

The psychometric tools that are used to measure traits in dogs and cats can be split into two main categories, based on their ultimate objectives and their authors’ academic field.

Firstly, we have tools that originate in the field of comparative psychology, which are primarily directed at exploring similarities in the higher order personality structure of people and animals. In people, the most widely accepted set of higher order personality traits are the big five [7]; extraversion (the tendency to experience positive emotions), neuroticism (the tendency to experience negative emotions), agreeableness (concerns for the needs of others), conscientiousness (ability to inhibit or constrain oneself, and follow premeditated goals), and openness (tendency to process abstract information flexibly and effectively) [12]. Recent research has gone beyond this to identify two meta-traits of stability and plasticity, that are derived from the big five, and even a single overall General Factor of Personality. Whether, and in what form, any of these constructs might be present in nonhuman species is arguable. For example, conscientiousness has only been found to exist as an independent trait in humans and chimpanzees [13]. These tools may be useful for scientific and exploratory purposes, but at this moment are of less relevance clinically.

Secondly, we have the more clinically-oriented tools that aim to identify underlying traits that could explain vulnerabilities and predispositions to certain behaviour problems. These usually follow a bottom-up approach that is focused on low-order traits such as impulse control, anxiousness, or aggressiveness. Examples include a scale developed to assess emotional predispositions in dogs (PANAS), a scale to assess attention and activity in dogs, and the wide range of battery tests aimed to identify temperament traits related to aggression [14,15,16,17]. Although the C-BARQ is regarded by its authors as a data collection tool, it does include subscales that, individually or in combination, provide information about traits such as excitability, anxiety and aggressiveness, and it has been used as the basis for a number of studies that have explored the biological basis of its subscales [10,18].

It is not clear whether the future of trait assessment lies in scales or battery tests. Owner-report questionnaires are potentially vulnerable to psychological biases and perception errors, but they do account for the dog’s behaviour in a wide range of situations, and over very long timescales. However, battery-tests are based on the dog’s reaction to a limited series of single events or situations [19,20] and may be more indicative of state than trait.

### 2.3. Axis 3: Health

This axis should evaluate the animal’s health along a continuum between excellent and poor health, using information about fundamental indicators of good health, such as exercise tolerance and body condition score, as well as specific information about any health problems the animal may be afflicted by. To be able to say that a dog is in excellent health, as opposed to not suffering from any known health problems, is of importance because it helps us to gauge the animal’s capacity for therapeutic activities, and can raise, or lower, the suspicion that that there are undetected health problems.

The association between behavioural problems and medical conditions can follow three main routes:

- A behaviour problem is the presenting complaint and a medical condition is subsequently identified as one of the contributory factors.
- A medical condition has presenting signs that are primarily behavioural (e.g., cognitive dysfunction, focal epilepsy, feline hyperesthesia syndrome).
- A medical condition is the presenting problem, but it could be partially caused by behaviour-related factors, including stress and certain behavioural traits. An example is the relationship between environmental stress and individual vulnerability to stress, and the occurrence of idiopathic cystitis in cats [21].

Apart from an estimation of general health, Axis 3 is primarily focused on the first and second routes, as these are typically presented in the behavioural medicine consultation.

If a patient is being evaluated for a behavioural problem, the connection between physical health and behaviour can be approached from a number of different perspectives:

- The animal has known health problems, but are they relevant to its current problem behaviour?
- The animal is receiving medication, supplements, nutraceuticals or a specialist diet, but are the effects, or adverse effects, of those biological therapies relevant to its current problem behaviour?
- The animal has no known health problems, but what level of suspicion is there that the current problem behaviour may be related to one or more undetected health problems?
- The animal has no known health problems, but is not in optimum health. Is that a contributory factor to the current behaviour, or an indication of other lifestyle factors that could be important?

Assessment of the health axis should start with a list of current and past health conditions, drug treatments, supplements, nutraceuticals, and diets. Diet is an emerging issue in behaviour, and there is increasing evidence about the influence of the gut microbiome on behaviour, with a recent study linking the composition of the gut flora with conspecific aggression in shelter dogs [22].

This list of conditions and treatments should be reviewed, to identify whether any of them might have effects that could be influencing the animal’s current behaviour, or may have influenced its development. There should then be a subjective evaluation of the animal’s overall health. 

Much like temperament traits, health problems introduce biases that alter the animal’s response to its environment in, among others, the following ways:

- Altering motivation (e.g., sickness behaviour, painful conditions, altered hunger, or thirst).
- Producing acute sensations that can alter behaviour (painful conditions, epilepsy).
- Causing functional impairment to sensory systems (e.g., touch, vision, or hearing).
- Causing functional impairment of the central nervous system, leading to perceptual, emotional, or cognitive impairment (e.g., brain tumour, cognitive dysfunction, epilepsy, hypothyroidism, or diabetes mellitus).

Health problems can have “deficit” and “productive” effects on behaviour:

- Deficit effects are characterised by a decrease in certain behaviours, such as activity, alertness, social interaction, feeding, and play.
- Productive effects are characterised by an increase in the expression of behaviours that were previously expressed at a lower level, and the appearance of behaviours that were previously absent. For example, activity, aggression, inappropriate elimination, eating, and self-mutilation.

The most common, and best understood, health problems that are associated with behavioural change in people and animals are; pain, sickness behaviour (resulting from inflammatory conditions and infection), epilepsy, and endocrine dysfunction. When assessing the medical axis for a case, we should consider whether any current behavioural signs, or changes in behaviour, might be associated with common conditions such as these.

Both chronic and acute pain are recognised as factors in defensive behaviour, and have been studied as part of the development of pain assessment tools [23,24]. In a study of health-related quality of life in dogs with chronic pain [24], owners of dogs with chronic pain reported changes in multiple aspects of their dogs’ behaviour, including sociability, demeanour, attention seeking, aggression, anxiety/fearfulness, ability to rest, and compulsive behaviour. More recently, an association has been found between orthopaedic pain and noise fear [25].

Sickness behaviour is a cytokine-mediated motivational state that results from infection and inflammation, in which individuals show a reduction in activity, sociability, play, and exploratory behaviour, and an increase in avoidance, social withdrawal, and sleep [26]. Sickness behaviour is not the result of a debilitated state, it is an active coping strategy that facilitates recovery and prevents disease transmission. Cytokines and sickness behaviour are also associated with depression [27], and anti-inflammatory drugs have been found to have beneficial effects on signs of depression when combined with antidepressant medication [28]. Given that sickness behaviour acts to generally reduce the expression of behaviour, we would expect that aggression might decrease when animals are in this state. This can create the paradoxical effect that recovery from inflammation, and sickness behaviour, can be associated with an increase, or return, of aggressive signs, particularly if pain is still present. Clearly, there is often an interaction between sickness behaviour and pain, as both are present together in many health problems.

Psychiatric comorbidities, including mood, anxiety, and psychotic disorders, are common in epilepsy, with these conditions occurring at rates 2-3-fold or higher in people with epilepsy than in the general population of people without epilepsy [29]. In human patients, focal seizures are more often associated with psychiatric comorbidity than are general seizures. Although research in companion animals is limited, there is some evidence to support some of the same associations. In a small study of abnormal behaviour in bull terriers [30], Dodman et al. found that tail chasing, irrational fear, and unprovoked aggression were associated with abnormal electro-encephalogram results. In a short case series, Stassen et al. identified paroxysmal episodes of acute fear and autonomic signs associated with focal seizures in Boerboel dogs with an onset of signs of three months [31]. In a survey of Groenendael and Tervueren dogs, 77.6% of those with epilepsy had focal seizures. Owners reported their dogs showed signs of depression up to several days before a seizure, and common behavioural signs associated with focal seizures included disorientation, attention seeking, and fearfulness [32]. In a retrospective study of behavioural changes associated with epilepsy, Shihab et al. found that in drug naive dogs, defensive aggression, fear and anxiety, and abnormal perception increased after epilepsy and there was a significant positive correlation between seizure frequency and score for defensive aggression [33]. In drug-treated dogs, there was an increase in fear and anxiety, abnormal perception, abnormal reactivity, attachment disorder, demented behaviour, and apathetic behaviour. The difference in behavioural changes between treated and untreated dogs may reflect a difference in severity or epilepsy, or a combination of epilepsy and iatrogenic, drug related, effects.

In humans, there is a link between endocrine disease and mental health problems [34]. Hypothyroidism has been recognised as having psychiatric manifestations since 1888. In people, it is most commonly associated with depression, delusions and hallucinations, and cognitive impairment. Other human psychiatric signs associated with endocrine disease are presented in Table 1.

Thyroid disease is common in companion animals and has been the focus of investigations to look for a connection with behaviour problems. In 2002, Fatjó et al. published a short case series of four dogs with aggression and hypothyroidism [35]. In all four cases, aggressive signs reduced with thyroid supplementation. However, in a cross-sectional study, Radosta et al. investigated the association between aggression and thyroid hormones but found no systematic relationship [36]. In a randomised, double-blinded, placebo-controlled study of the effect of thyroid replacement therapy on aggressive dogs (owner-directed) with suboptimal thyroid function, Dodman et al. did not find an effect [37]. Hrovat et al. tracked behavioural changes during levothyroxine therapy over six months in clinically hypothyroid dogs using the C-BARQ questionnaire, and found no change other than an increase in the score for the activity sub-scale [38]. Taken as a whole, this suggests that, as in human medicine, clinical hypothyroidism may be associated with behavioural pathology in individual cases, but that thyroid function does not have a generalised effect on aggression.

Hyperadrenocorticism is associated with a wide range of psychiatric signs in people, including anxiety, panic, mania, mood disorders, and depression. Although this has not been studied in dogs, behavioural change is commonly identified as an early indicator of the disease. Iatrogenic effects of cortisol administration have been reported, and include reduced playfulness, increased fearfulness and restlessness, and aggression around food [39,40].

Feline hyperesthesia syndrome (FHS) and feline interstitial cystitis (FIC), are physical health problems with a primary behavioural manifestation [26]. FIC is most commonly associated with problems of inappropriate elimination, but it can be connected with other problems such as fearful avoidance or owner-directed aggression if the owner is confrontational or punitive. FHS is associated with owner directed aggression and inter-cat aggression. Both are conditions that may not be apparent from the behavioural or clinical history, unless attention is paid to the details of incidents and their pattern.

One of the main challenges for the veterinary behaviourist is to decide the level of clinical investigation that is required in each case. If there is a strong suspicion that any of the above conditions are contributing to the animal’s behaviour, this could provide justification for further investigation. If not, then a useful set of indicators that suggest that further analysis, or screening, for undiagnosed medical conditions could include [41]:

- In cases where it is difficult to establish a link between environmental cues and the behaviour of the animal.
- When the first signs of behavioural problem are seen in adulthood.
- If the patient is middle aged or older, they are more likely to have a physical condition.
- A period of sub-optimal health precedes the behavioural signs.
- There are neurological signs.
- There are signs of weight loss.
- There are heritable disorders in the animal’s family, line, or breed.
- There have been signs of changing level of consciousness or awareness, or other signs of mental impairment.
- There are some physical signs that are indicative of serious physical disease (fever, abdominal swelling, oedema, jaundice, and pain).
- Behavioural signs do not resolve, despite appropriate treatment.
- There is evidence of generally worsening physical health (e.g., exercise intolerance).
- The absence, or reduced expression, of species-typical behaviours, such as exploration, play, social interaction, or locomotion (e.g., climbing or jumping), particularly when there has been a change.

Once a basic medical examination and assessment have been conducted, information from Axis 1 could help the clinician to decide what additional medical tests could be helpful.

### 2.4. Axis 4: Environment

As with health, the aim of this axis is not only to identify any environmental deficiencies that could contribute to behavioural problems, but also to consider where the environment is located along a continuum from excellent to poor quality. An excellent environment is one that offers the animal an opportunity for a “good life”, as we discuss in the section on Axis 5 (functioning). An assessment of the environment axis should include an evaluation of:

- The quality of the physical environment.
- The quality of the social environment.
- Predictability and control, from the animal’s perspective.
- The individual’s preparedness for, and ability to utilise, the environment (which may relate to the individual’s temperament, state of health, and previous environmental experience).

Codes for the welfare of companion animal species already exist at national level within the European Union (EU), as part of EU welfare legislation; these codes provide information about the needs of those species and how to provide for them.

To assess the quality of the animal’s physical and social environment, it should be compared with the set of needs in the relevant code, with the aim of quantifying:

- How well the animal’s current environment, satisfies the needs outlined in the relevant welfare code for that species.
- How well the animal’s current environment provides suitable opportunities for the animal to manage its own negative emotions and stress (e.g., places to withdraw to, in order to avoid fear or conflict).

These points should be evaluated with respect to any specific physical limitations or health problems, such as sensory loss or osteoarthritis (identified in Axis 3), that might impair the individual’s ability to fully utilise its environment. Such limitations may lead to welfare issues and negative emotional states, such as frustration.

Apart from the amount of resources, such as food and resting places, that are available, there is also the matter of control over how those resources are accessed. For example, whether the animal is meal fed, fed on demand, or given ad libitum access to food. Meal frequency in cats may be around 12 times per day [42], so ad-libitum access to food would be preferable to twice daily meal feeding as it provides the cat with greater control. Control over access to places to hide from or avoid sources of fear, such as unfamiliar people loud noises, may be particularly important.

Predictability of the environment is also important. In a laboratory study of the effects of an unexpected environmental event (UEE), including failure of light timers and temperature regulation, changes in care-taking personnel, intermittent loud noises, and movement of cats between rooms and cages, Stella et al. found that cats that were exposed to UEE (compared with control cats with a normal environmental schedule) were 9.3 times more likely not to eat or eliminate in a 24 h period, 9.8 times more likely to defecate outside the litter-box, and 1.6 times more likely to urinate outside the litter-box [43]. There were no differences in the level of environmental enrichment between the group with UEE and the group with a normal environmental schedule. In a similar controlled study by Carlstead et al., cats that were exposed to a 21-day period of irregular feeding and cleaning times, absence of talking and petting by humans, and daily unpredictable manipulations, showed significantly raised urinary cortisol, increased hiding and vigilance, and reduced exploratory behaviour [44]. This research suggests that, at least in cats, an unpredictable environment can, irrespective of the quality of the environment, lead to stress and problem behaviour.

The owner is part of the animal’s social environment as a source of security, a means of access to resources, and a source of stimulation. However, owners can also become a significant source of distress, as a consequence of how they interact with their pets. For instance, the use of punishment has been associated to a higher prevalence of behaviour problems in dogs [45,46].

An owner’s personality factors may influence the way they interact with the dog [47]; for instance, extraverted owners tend to praise their dogs more. Owner personality and psychological factors seem to influence the expression of behaviour problems in their pets. Personality traits such as neuroticism or emotional instability, as well as psychiatric conditions such as post-traumatic stress disorder, have been considered risk factors for dog behaviour problems, including aggression and separation anxiety [46].

According to the secure base effect, which is similar to that observed in children [48], the individual exhibits a wider range of behaviours from its repertoire, including play and exploration, when it is in the presence of the secure base (a familiar individual). In a study of the problem-solving behaviour of dogs, Horn et al. found that dogs interacted with the apparatus for longer if they were in the presence of their owner, but the same effect was not observed with an unfamiliar individual [48].

The secure base effect may influence the degree to which dogs interact with their environment, even if it is highly enriched, when the owner is absent. Dogs that live in family environments, with multiple people who can provide a secure base for them, may be better able to cope than those with a single attachment figure. Evidence for this is provided by a study of risk factors for separation anxiety in dogs [49], which found that dogs that were kept by a single person were 2.5 times more likely to experience separation anxiety. Apart from the presence of the owner, the dog’s attachment style can influence the animal’s behaviour. Dogs that have an avoidant attachment style have been found to have a higher risk of developing separation-related disorders [50].

How an animal responds to an environment, and whether it will be able to make use of the opportunities to express normal behaviour and exert control over negative emotions and stress, depends on other factors. Two that have already been mentioned are unpredictability of the environment (which can lead to hiding and reduced exploratory behaviour), and the absence of a secure base (which reduces also exploration, play, and social engagement).

In addition, an animal’s familiarity with the type of environment, and its temperament, will affect its response to the environment. An animal that has been reared in a nondomestic environment, or has lived in an impoverished environment for an extended period of time, may find it difficult to engage with its environment and utilise the resources that are available to it, even if the environment is highly enriched. Likewise, an animal with anxious or avoidant traits. Therefore, there are links between the environment axis and other axes, such as traits.

The environment constrains an animal’s coping and behavioural responses, producing a degree of consistency in responses that could resemble a trait. This is true even when the environment is unpredictable; the animal still has to adopt a consistent strategy for coping with such an environment. The most extreme example of this is an environment that includes unpredictable, unavoidable, strongly aversive events. In one of the first studies to demonstrate this, Overmier and Seligman found that dogs that were subjected to unpredictable, unavoidable shock entered a persistent state of learned helplessness [51].

### 2.5. Axis 5: Functioning

In this axis, the objective is to assess the animal’s ability to function in its environment, and as a pet. Primarily, this means evaluating the animal’s quality of life and the human-animal bond, including both the positive and negative impacts that the animal has on its owners’ lifestyle. As with health and environment, this should not concentrate on sources of problems, it should also include information about success or achievement. For example, the dog having a wide social group, successfully participating in activities such as agility training, or providing the owner with pleasure and support. This is because these successes and achievements give an indication of where the animal’s positive motivations lie, and how its quality of life and its relationship with the owner might be improved.

### 2.6. The Animal’s Quality of Life

The concept of animal welfare previously focused on the absence of indicators, and sources, of stress and emotional discomfort. This was typified by the Five Freedoms Paradigm that was introduced in the early 1990s by John Webster for farm animals, and later adapted for companion animals [52]. This paradigm was biased towards the identification of negative indicators (of stress and lack of wellbeing) but it is still a useful checklist with which to assess the provision of care an animal is receiving. It defines a baseline of minimum requirements, below which the animal’s welfare would be impaired, and above which positive wellbeing could be experienced by the animal.

For an individual to experience genuine wellbeing, as opposed to the lack of a welfare problem, there has to be the opportunity for the expression of positive emotional states. In other words, wellbeing is not only about avoiding things that the animal dislikes, or meeting minimum requirements for the expression of normal behaviour, but is also about being able to do things that are enjoyable. Indeed, the so-called behavioural needs are defined as behaviours linked to positive emotional states that the animal should be given the opportunity to express on a regular basis in order to have wellbeing. Examples of these positive emotional states in dogs and cats include exploration of a complex environment, play, and social interaction.

This, more recently developed, concept of wellbeing, has led to a paradigm shift toward “Quality of Life” and “A life worth living” [52,53]. According to the model developed by Mellor [52], an individual’s quality of life can be placed into one of the following categories:

- A good life: The balance between positive and negative experiences and affective states is clearly positive.
- A life worth living: The net balance between positive and negative experiences and affective states is still satisfactory, but less so.
- A point of balance: Positive and negative experiences and affective states are equally balanced.
- A life worth avoiding: The balance between positive and negative experiences and affective states starts to be unfavourable.
- A life not worth living: The balance between positive and negative experiences and affective states is strongly unfavourable.

Quality of life can be seen as the endpoint of all the factors included in the five freedoms paradigm. In other words, nutrition, physical health, stress, and positive behavioural expression all contribute to the emotional state the animal experiences in one particular moment.

In a clinical setting, this could provide the basis for an informal, but useful, tool for rating the overall quality of life of veterinary patients, including those with behavioural problems, which could be evaluated by summing wellbeing evaluations in a range of typical situations experienced by the dog as part of its regular routine.

A behaviour problem itself can impair the animal’s quality of life through different mechanisms:

- By increasing the likelihood of punishment.
- By damaging the secure-base relationship.
- By imposing restrictions on the physical environment; for example, dogs that exhibit destructive behaviour are often confined to certain areas of the house.
- By limiting opportunities to explore the environment and to interact with people and other animals; for example, problems of aggression often result in confinement and/or on the application of restraint methods during walks.
- By increasing the risk of relinquishment and euthanasia; for example, behaviour problems are known to be the most common reason for owners to surrender their dogs to shelters [46].

On a moral basis, improving the animal’s quality of life is, in itself, a worthwhile objective that we have a professional duty to achieve. However, on a pragmatic basis, it is also important to address welfare as animals that are chronically stressed or frustrated are less likely to respond well to therapy.

### 2.7. The Family’s Quality of Life

Although living with a pet is usually linked to psychological and physical wellbeing, it can negatively impair the family’s quality of life. Benefits of pet ownership include providing emotional support, opportunities for enjoyable interactions, and opportunities to develop shared activities. Perceived costs of pet ownership include restrictions imposed on lifestyle, changes in the social network, and financial constraints [54].

The social-exchange theory is a good model to understand the balance between the positive and negative aspects of a relationship [55]. According to it, a relationship is considered to be successful if there is a positive balance between its associated benefits and costs. If a relationship’s costs outweigh its rewards, then the person would be more willing to terminate it.

Following this theoretical framework, psychometric scales have been developed for owners of dogs and cats, that are able to quantify the three main dimensions of the human–animal bond: The pattern of interaction, emotional closeness, and the perceived cost [55,56]. From a practical point of view, it is important to note that emotional closeness is the most influential dimension on the overall quality of the relationship [54]. In other words, if the owner is highly satisfied about the positive elements of the relationship, the negative impact of a behaviour problem will be perceived to be reduced. Otherwise, relationships between people and their pets would collapse more often, resulting in relinquishment or euthanasia.

A behaviour problem can negatively impact a human–animal relationship in two different ways: First, by increasing the perceived cost of the relationship, and second, by impairing the emotional bond between the family and the pet. In preliminary work by the authors, we found an association between impact on owner lifestyle, as measured with MDORS, and problem behaviour, using C-BARQ; dog directed aggression, separation problems, and training issues (miscellaneous problems) were all associated with a negative impact on owner lifestyle (perceived costs). This work has been followed up by Herwijnen et al. who found similar relationships between aggression and disobedience, and perceived costs and satisfaction with ownership [57]. However, those authors did not examine the relationship between MDORS and a wide range of other behaviour problems.

From a clinical perspective, it is important to estimate the severity of a behaviour problem, not only according to its objective features, but also by its impact on the family’s quality of life. For many cases, the primary presenting problem may be objectively less serious than other problems the animal shows, but nevertheless they are the owner’s focus. Axis 1 and Axis 5 complement each other, with the former being focused on the objective description of the animal’s behavioural profile and the latter on the owner’s perception and the costs of ownership they perceive.

The treatment of a behaviour problem can itself become a perceived cost, not only for financial reasons, but also because it is time consuming and very often challenges the way people interact with their pets. In addition to the financial cost of treatment, performing behavioural therapy places a time penalty on the owner; together, these combined costs may have a substantial impact on the owner’s quality of life.

Although scales measuring the human–animal bond constitute useful tools to research human–animal relationships, there are still no reference values that could be used by clinicians to assess one particular relationship. However, the use of these scales still provides a valuable tool for evaluating the current relationship, potential sources of concern, and improvements that might result from the treatment of the behavioural problem. The study of owner–dog relationship profiles offers an alternative method to study the costs and negative aspects of dog ownership, and thereby to bring to light important factors contributing to a successful owner–dog relationship.

Finally, it should be remembered that behaviour problems can also have an impact on the community’s quality of life. Examples include dog bites occurring in public spaces, as well as the contribution of dog and cat vocalizations to noise pollution [58].

## 3. Discussion and Conclusions

The current classification systems used in veterinary behavioural medicine are facing the same challenges that have already been faced in human mental health. Therefore, it is worth considering an overview of the past and current direction of research and clinical practice in human mental health, as an indication of the direction that veterinary behavioural medicine ought to be following.

In human mental health, the gold standards for diagnostic classification have been the Diagnostic and Statistical Manual of Mental Disorders (DSM) and the International Classification of Diseases (ICD). These formalised systems were used to provide patients with specific diagnoses, which could then be used within the healthcare and health insurance framework.

There are two key features to describe DSM and ICD. First, they try to provide a list of specific diagnostic categories, each of them linked to a relatively well-defined list of signs and risk factors. Second, they propose a multi-axial assessment that forces the clinician to consider a broad range of information, including medical, psychosocial, and cultural influences.

Diagnoses in the two systems were very similar. Although, throughout its history, DSM has provided lists of necessary and sufficient criteria for each diagnosis, until recently the ICD has just provided a list of descriptive titles and no specific guidance on diagnosis [59]. The aim of the DSM, in particular, was to improve consistency and reliability of diagnoses, ensuring that any well-trained mental health professional could reach a diagnosis, regardless of their theoretical orientation.

However, the DSM and ICD systems have been unable to integrate basic research that relates to the common dimensional characteristics that are thought to underpin many mental health problems. Attempts to revise the DSM, for edition five, to provide a stronger biological underpinning for its classification system, have failed because biological factors not only cut across multiple disorders, but also varied within them [60].

As a result of the weaknesses in the current clinical classification models, and, in particular, the lack of progress in understanding the aetiology of mental health disorders, the National Institute of Mental Health (NIMH) has abandoned funding research using the DSM/ICD classification systems, and has instead proposed a new path for mental health research, based on Research Domain Criteria (RDoC) [61].

The top-down approach of DSM/ICD has been replaced with a bottom-up approach that focusses on the basic behavioural and brain mechanisms that are involved in psychopathology. The aim is to identify transdiagnostic mechanisms that will more likely have a specific aetiology, biological and genetic basis, in order to find new therapeutic opportunities.

Two of the four strategic goals of the RDoC approach are a commitment to studying the “full range of variation, from normal to abnormal” [62] and to “Integrate the fundamental genetic, neurobiological, behavioural, environmental, and experiential components that comprise these mental disorders” [62].

Whilst incompatible with classification models, all of the strategic goals are highly compatible with revised multi-axial assessment models of the type that are present in DSM/ICD, and that we are now proposing for veterinary behavioural medicine. The RDoC research framework defines a set of domains of functioning. Within each domain are a number of constructs; behavioural elements, processes, mechanisms, and responses, that comprise different aspects of the overall range of functions within that domain. The intention is that constructs should be studied across their full range of functioning from normal to abnormal, with the understanding that each is situated in, and affected by, environmental and neurodevelopmental contexts. Table 2 summarises the current list of domains and constructs (for a full description, consult the NIMH website, as RDoC is subject to regular updates).

For each construct, there may be a set of subconstructs. For example, within “motor actions” there are subconstructs of action planning and selection, sensorimotor dynamics, initiation, execution, and inhibition and termination.

Due to the very basic level of the domains, constructs and subconstructs, the vast majority are as relevant to veterinary behavioural medicine as they are in human mental health. Indeed, many of them are immediately recognisable as the types of characteristics we observe when we are assessing cases. This provides an excellent basis for comparative studies between behavioural and mental health problems in human and veterinary species.

The range of conditions is currently much smaller in veterinary behaviour than human mental health, and this may reflect the presence of a narrower set of underlying psychopathological processes. However, in the absence of reported phenomenology, we may simply be unaware of some aspects of the psychopathology of our veterinary patients.

We propose that veterinary behavioural medicine should parallel developments in the approach to human mental health, as set out by the NIMH, for several reasons:

- Transdiagnostic mechanisms are likely to be shared between species: Developments in human mental health could therefore enable us to identify treatable features of behavioural problems that we are currently unaware of.
- New biological tests and therapies for human mental health problems will target transdiagnostic mechanisms: If we work in parallel with current developments in human mental health, and adopt a similar theoretical framework, it will be easier to make rational use of those new tests and therapies.
- Veterinary behavioural problems such as compulsive disorder, phobia, anxiety, and aggression, share common characteristics with human conditions: They offer excellent opportunities for research funding within the RDoC framework.

A major advantage in veterinary behavioural medicine is that we have a greater opportunity to collect information about basic aspects of behavioural responses, and we often have much more complete information about the rearing environment, life experiences, and lifestyle of our patients than is possible for human patients. A major disadvantage is lack of a formal framework for collecting that information.

The approach that we propose represents a practical way to structure the collection of detailed information about all aspects of a case, organise that information into five separate axes, maintaining the equality of importance of those independent axes, but at the same time forcing us to critically consider the interaction between them. Rather than limiting researchers and clinicians to a prescriptive set of diagnoses and tests, it encourages flexibility, points to potential areas of research, and is easily able to accommodate new information, particularly on the aetiology of disorders, as this becomes available.

However, this model needs to be subjected to a process of development and validation. Whilst setting out this task is beyond the scope of this paper, we can offer some comments. The process could include confirmation of the optimal overall structure of the model, including the number of axes and their content, the development of specific tools to be used to assess features within each axis (such as scales and tests of temperament), and testing of the clinical applicability of the model.

## Figures and Tables

**Table 1:** Psychiatric signs associated with endocrine disease in human patients (after, Conner and Solomon [34]).

Endocrine Condition	Psychiatric Symptoms (Human)
Hyperadrenocorticism	Anxiety, Panic disorders, Irritability, Mania, Hypomania, Poor self-image hypercortisolism irritability, Distress, Mood disorders, Depression.
Type 1 Diabetes	Depression, Psychomotor agitation, Sleeping difficulty, Eating disorders.
Type 2 Diabetes	Depression, Eating disorders, Poor self-image.
Hyperthyroidism	Anxiety, Irritability, Mania, Hypomania, Psychosis, Insomnia, Attention and Overactivity problems, Depression, Restlessness, Fatigue, and Delirium.
Hypothyroidism	Delusions, hallucinations, attention deficits and cognitive disturbances.
Pheochromocytoma	Anxiety, Panic disorders, Tremulousness.
Hypoparathyroidism	Depression.
Androgens	Increased aggression, Anger, Acting out, Dominant behaviour, Antisocial behaviour.

**Table 2:** Current list of NIMH RDoC domains and constructs.

Domain	Constructs					
Negative valence systems	Acute threat (fear)	Potential threat (anxiety)	Sustained threat	Loss	Frustrative nonreward	
Positive valence systems	Reward responsiveness	Reward learning	Reward valuation			
Cognitive systems	Attention	Perception	Declarative memory	Language	Cognitive control	Working memory
Social processes	Affiliation and attachment	Social communication	Perception and understanding of self	Perception and understanding of others		
Arousal and regulatory systems	Arousal	Circadian rhythms	Sleep and wakefulness			
Sensorimotor systems.	Motor actions	Agency and ownership	Habit	Innate motor patterns		

## References

[1] Landsberg G., Pageat P., Fatjó J., Landsberg G., Hunthausen W., Ackerman L. (2013). Terminology, pathology and the Pageat (French) approach to behavioral disorders. Behavior Problems of the Dog and Cat.

[2] Sheppard G., Mills D.S. (2003). Construct models in veterinary behavioural medicine: Lessons from the human experience. Vet. Res. Commun..

[3] Westgarth C. (2015). Why nobody will ever agree about dominance in dogs. J. Vet. Behav..

[4] Kring A.M., Johnson S.L. (2013). Diagnosis and Assessment. Abnormal Psychology.

[5] Hsu Y., Serpell J.A. (2003). Development and validation of a questionnaire for measuring behavior and temperament traits in pet dogs. J. Am. Vet. Med. Assoc..

[6] Duffy D.L., Diniz de Moura R.T., Serpell J.A. (2017). Development and evaluation of the Fe-BARQ: A new survey instrument for measuring behavior in domestic cats (*Felis s. catus*). Behav. Process..

[7] Shiner R., DeYoung C., Zelazo P.D. (2011). The Structure of Temperament and Personality Traits: A Developmental Perspective. The Oxford Handbook of Developmental Psychology.

[8] Rosado B., García-Belenguer S., León M., Chacón G., Villegas A., Palacio J. (2011). Effect of fluoxetine on blood concentrations of serotonin, cortisol and dehydroepiandrosterone in canine aggression. J. Vet. Pharm. Ther..

[9] Bray E.E., MacLean E.L., Hare B.A. (2015). Increasing arousal enhances inhibitory control in calm but not excitable dogs. Anim. Cogn..

[10] Bowen J., Fatjó J., Serpell J.A., Bulbena-Cabré A., Leighton E., Bulbena A. (2019). First evidence for an association between joint hypermobility and excitability in a non-human species, the domestic dog. Sci. Rep..

[11] Wright H.F., Mills D.S., Pollux P.M.J. (2011). Development and validation of a psychometric tool for testing impulsivity in the domestic dog (Canis familiaris). Int. J. Comp. Psychol..

[12] DeYoung C.G., Hirsh J.B., Shane M.S., Papademetris X., Rajeevan N., Gray J.R. (2010). Testing Predictions From Personality Neuroscience: Brain Structure and the Big Five. Psychol. Sci..

[13] Gosling S.D., John O.P. (1999). Personality Dimensions in Nonhuman Animals: A Cross-Species Review. Curr. Dir. Psychol. Sci..

[14] Vas J., Topál J., Péch E., Miklósi A. (2007). Measuring attention deficit and activity in dogs: A new application and validation of a human ADHD questionnaire. Appl. Anim. Behav. Sci..

[15] Sheppard G., Mills D.S. (2002). The Development of a Psychometric Scale for the Evaluation of the Emotional Predispositions of Pet Dogs. Int. J. Comp. Psychol..

[16] Patronek G.J., Bradley J. (2016). No Better Than Flipping a Coin: Reconsidering Canine Behavior Evaluations in Animal Shelters. J. Vet. Behav..

[17] Netto W.J., Planta D.J.U. (1997). Behavioural testing for aggression in the domestic dog. Appl. Anim. Behav. Sci..

[18] Liinamo A., van den Berg L., Leegwater P.A., Schilder M.B.H., van Arendonk J.A.M. (2006). Genetic variation in aggression-related traits in Golden Retriever dogs. Appl. Anim. Behav. Sci..

[19] Miklosi A. (2015). The organisation of individual behaviour. In Dog Behaviour, Evolution, and Cognition.

[20] Wiener P., Haskell M.J. (2016). Use of questionnaire-based data to assess dog personality. J. Vet. Behav..

[21] Lund H.S., Saevik B.K., Oystein W.F., Grontvedt E.T., Vatne T., Eggertsdóttir A.V. (2019). Risk factors for idiopathic cystitis in Norwegian cats: A matched case-control study. J. Feline Med. Surg.

[22] Kirchoff N.S., Udell M., Sharpton T.J. (2019). The gut microbiome correlates with conspecific aggression in a small population of rescued dogs (*Canis familiaris*). PeerJ.

[23] Holton L., Reid J., Scott E.M., Pawson P., Nolan A. (2001). Development of a behaviour-based scale to measure acute pain in dogs. Vet. Rec..

[24] Wiseman-Orr M.L., Nolan A.M., Reid J., Scott E.M. (2004). Development of a questionnaire to measure the effects of chronic pain on health-related quality of life in dogs. Am. J. Vet. Res..

[25] Fagundes A.L.L., Hewison L., McPeake K.J., Helen Zulch H., Mills D.S. (2018). Noise Sensitivities in Dogs: An Exploration of Signs in Dogs with and without Musculoskeletal Pain Using Qualitative Content Analysis. Front. Vet. Sci..

[26] Fatjó J., Bowen J.E., Horwitz D.F., Mills D.S. (2009). Medical and metabolic influences on behavioural disorders. BSAVA Manual of Canine and Feline Behavioural Medicine.

[27] Danzer R. (2009). Cytokine, Sickness Behavior, and Depression. Immunol Allergy Clin North. Am..

[28] Müller N., Schwarz M.J., Dehning S., Douhe A., Cerovecki A., Goldstein-Müller B., Spellmann I., Hetzel G., Maino K., Kleindienst N. (2006). The cyclooxygenase-2 inhibitor celecoxib has therapeutic effects in major depression: Results of a double-blind, randomized, placebo controlled, add-on pilot study to reboxetine. Nat. Mol. Psychiatry.

[29] Josephson C.B., Jetté N. (2017). Psychiatric comorbidities in epilepsy. Int. Rev. Psychiatry.

[30] Dodman N.H., Knowles K.E., Shuster L., Moon-Fanelli A.A., Tidwell A.S., Keen C.L. (1996). Behavioral changes associated with suspected complex partial seizures in bull terriers. J. Am. Vet. Med. Assoc..

[31] Stassen Q.E.M., Grinwis G.C.M., van Rhijn N.C., Beukers M., Verhoeven-Duif N.M., Leegwater P.A.J. (2019). Focal epilepsy with fear-related behavior as primary presentation in Boerboel dogs. J. Vet. Intern. Med..

[32] Berendt M., Gulløv C.H., Christensen S.L.K., Gudmundsdottir H., Gredal H., Fredholm M., Alban L. (2008). Prevalence and characteristics of epilepsy in the Belgian shepherd variants Groenendael and Tervueren born in Denmark 1995–2004. Acta Vet. Scand..

[33] Shihab N., Bowen J., Volk H.A. (2011). Behavioral changes in dogs associated with the development of idiopathic epilepsy. Epilepsy. Behav..

[34] Conner S.H., Solomon S.S. (2017). Psychiatric Manifestations of Endocrine Disorders. J. Hum. Endocrinol..

[35] Fatjo J., Stub C., Manteca X. (2002). Four cases of aggression and hypothyroidism in dogs. Vet. Rec..

[36] Radosta L.A., Shofer F.S., Reisner I.R. (2012). Comparison of thyroid analytes in dogs aggressive to familiar people and in non-aggressive dogs. Vet. J..

[37] Dodman N.H., Aronson L., Cottam N., Dodds J.W. (2013). The effect of thyroid replacement in dogs with suboptimal thyroid function on owner-directed aggression: A randomized, double-blind, placebo-controlled clinical trial. J. Vet. Behav..

[38] Hrovat A., De Keuster T., Kooistra H.S., Duchateau L., Oyama M.A., Peremans K., Daminet S. (2019). Behavior in dogs with spontaneous hypothyroidism during treatment with levothyroxine. J. Vet. Intern. Med..

[39] Notari L., Burman O., Mills D. (2015). Behavioural changes in dogs treated with corticosteroids. Physiol. Behav..

[40] Notari L., Burman O., Mills D. (2016). Is there a link between treatments with exogenous corticosteroids and dog behaviour problems?. Vet. Rec..

[41] Morrison J. (2015). When Psychological Problems Mask Medical Disorders.

[42] Houpt K.A. (1991). Domestic Animal Behavior.

[43] Stella J.L., Lord L.K., Buffington C.A. (2011). Sickness behaviours in response to unusual external events in healthy cats and cats with FIC. J. Am. Vet. Med. Assoc..

[44] Carlstead K., Brown J., Strawn W. (1993). Behavioural & physical correlates of stress in laboratory cats. Appl. Anim. Behav. Sci..

[45] Hiby E.F., Rooney N.J., Bradshaw J.W.S. (2004). Dog training methods: Their use, effectiveness and interaction with behaviour and welfare. Anim. Welf..

[46] Dodman N.H., Brown D.C., Serpell J.A. (2018). Associations between owner personality and psychological status and the prevalence of canine behavior problems. PLoS ONE.

[47] Kis A., Turcsán B., Miklósi A., Gácsi M. (2012). The effect of the owner’s personality on the behaviour of owner-dog dyads. Interact. Stud..

[48] Horn L., Huber L., Range F. (2013). The Importance of the Secure Base Effect for Domestic Dogs—Evidence from a Manipulative Problem-Solving Task. PLoS ONE.

[49] Flannigan G., Dodman N.H. (2001). Risk factors and behaviors associated with separation anxiety in dogs. JAVMA.

[50] Konok V., Kosztolányi A., Rainer W., Mutschler B., Halsband U., Miklósi A. (2015). Influence of Owners’ Attachment Style and Personality on Their Dogs’ (*Canis familiaris*) Separation-Related Disorder. PLoS ONE.

[51] Overmier J.B., Seligman M.E.P. (1967). Effects of inescapable shock upon subsequent escape and avoidance learning. J. Comp. Physiol. Psychol..

[52] Mellor D.J. (2016). Updating Animal Welfare Thinking: Moving beyond the “Five Freedoms” towards “A Life Worth Living”. Animals.

[53] McMillan F.D. (2000). Quality of life in animals. JAVMA.

[54] Calvo P., Bowen J., Bulbena A., Tobeña A., Fatjó J. (2016). Highly Educated Men Establish Strong Emotional Links with Their Dogs: A Study with Monash Dog Owner Relationship Scale (MDORS) in Committed Spanish Dog Owners. PLoS ONE.

[55] Dwyer F., Bennett P.C., Coleman G.J. (2006). Development of the Monash Dog Owner Relationship Scale (MDORS). Anthrozoos.

[56] Howell T.J., Bowen J., Fatjó J., Calvo P., Holloway A., Bennett P.C. (2017). Development of the cat-owner relationship scale (CORS). Behav. Process..

[57] Herwijnen I.R.V., van der Borg J.A.M., Naguib M., Beerda B. (2018). Dog ownership satisfaction determinants in the owner-dog relationship and the dog’s behaviour. PLoS ONE.

[58] Utley W.A., Buller I. (1988). B A study of complaints about noise from domestic premises. J. Sound Vib..

[59] Clark L.A., Cuthbert B., Lewis-Fernández R., Narrow W.E., Reed G.M. (2017). Three Approaches to Understanding and Classifying Mental Disorder: ICD-11, DSM-5, and the National Institute of Mental Health’s Research Domain Criteria (RDoC). Psychol. Sci. Publ. Int..

[60] Hyman S.E. (2007). Can neuroscience be integrated into the DSM-V?. Nat. Rev. NeuroSci..

[61] Cuthbert B.N. (2017). Research Domain Criteria: Toward future psychiatric nosologies. Dialogues Clin. NeuroSci..

[62] Cuthbert B.N., Insel T.R. (2013). Toward the future of psychiatric diagnosis: The seven pillars of RDoC. BMC Med..

